# Practical Guide to Interpreting Cardiac Magnetic Resonance in Patients with Cardiac Masses

**DOI:** 10.3390/jcdd10060229

**Published:** 2023-05-24

**Authors:** Giulia Grazzini, Silvia Pradella, Alice Rossi, Rocco Pio Basile, Matteo Ruggieri, Daniele Galli, Anna Palmisano, Pierpaolo Palumbo, Antonio Esposito, Vittorio Miele

**Affiliations:** 1 Department of Emergency Radiology, University Hospital Careggi, Largo Brambilla 3, 50134 Florence, Italy; pradella3@yahoo.it (S.P.); alicerossi.ar@gmail.com (A.R.); roccopiobasile@gmail.com (R.P.B.); mruggieri00@gmail.com (M.R.); dangalli91@gmail.com (D.G.); vmiele@sirm.org (V.M.); 2 Experimental Imaging Center, San Raffaele Scientific Institute, Via Olgettina 60, 20100 Milan, Italy; palmisano.anna@hsr.it (A.P.); esposito.antonio@hsr.it (A.E.); 3 School of Medicine, Vita-Salute San Raffaele University, Via Olgettina 58, 20132 Milan, Italy; 4 Department of Biotechnological and Applied Clinical Sciences, University of L’Aquila, Via Vetoio 1, 67100 L’Aquila, Italy; palumbopierpaolo89@gmail.com

**Keywords:** cardiac mass, cardiac magnetic resonance, imaging, tissue characterization

## Abstract

It is common for a cardiac mass to be discovered accidentally during an echocardiographic examination. Following the relief of a cardiac mass, being able to evaluate and characterize it using non-invasive imaging methods is critical. Echocardiography, computed tomography (CT), cardiac magnetic resonance imaging (CMR), and positron emission tomography (PET) are the main imaging modalities used to evaluate cardiac masses. Although multimodal imaging often allows for a better assessment, CMR is the best technique for the non-invasive characterization of tissues, as the different MR sequences help in the diagnosis of cardiac masses. This article provides detailed descriptions of each CMR sequence employed in the evaluation of cardiac masses, underlining the potential information it can provide. The description in the individual sequences provides useful guidance to the radiologist in performing the examination.

## 1. Introduction

A cardiac mass is commonly discovered as an accidental discovery during an echocardiographic examination performed for identifying other causes [1]. The most common type of cardiac mass is “pseudotumors,” such as an intracardiac thrombus or misinterpreted normal anatomic variants [2,3]. Heart tumors are rare and often benign [4,5,6]. The accurate characterization of cardiac masses is key in the management of these patients. Radiological imaging serves a crucial role in the evaluation of cardiac masses. Several imaging modalities can be used to evaluate cardiac masses: echocardiography, computed tomography (CT), magnetic resonance imaging (MRI), and positron emission tomography (PET) [1,7,8,9,10]. The imaging modality used for evaluating cardiac masses depends on several factors, including the suspected diagnosis, the location and characteristics of the mass, and the patient’s clinical status. Nonetheless, echocardiography is often the first-line imaging modality, while CT, MRI, and PET scans may be used for further evaluation in certain cases. In particular, cardiac magnetic resonance (CMR) is the best technique for the non-invasive characterization of tissues.

This article provides detailed descriptions of each CMR sequence that allow the most favorable characterization of cardiac masses in order to facilitate their differential diagnosis. The authors have selected some cases from the archive with histological findings and CMR to identify specific characteristics of the most and least common lesions. Compared to other studies available in the literature, for each sequence used, the potential information it can provide has been identified. The description of the individual sequences’ usefulness can guide the radiologist in performing the examination.

## 2. Epidemiology

Epidemiologic data, based on autopsy series, indicate primary cardiac tumors (PCTs) to have an incidence of 0.002–0.3% and a prevalence of 0.001–0.03% [2,11]. Indeed, considering the rarity of these tumors, the percentage of malignant masses in the various reported series is also highly variable [11]. In their meta-analysis, Paraskevaidis et al. demonstrated that benign PCTs represent the most prevalent form of cardiac tumors, while malignant PCTs and secondary malignant cardiac tumors (SMCTs) each represent 10% of all cardiac tumors, in contrast with previous, outdated studies reporting the incidence of SMCTs to be at least 100 times higher than that of PCTs [8].

The prevalence rates of cardiac tumors significantly differed based on age, group, and sex [12,13,14]. Reflecting the age distribution of patients with malignant diseases, cardiac metastases predominantly occur in patients in the sixth and seventh decade of their life, and in this case, there is no sex preference [2,6].

Theoretically, every malignant tumor can metastasize to the heart. To date, only tumors of the central nervous system have not been proven to develop cardiac metastases. Owing to their high propensity for generalized hematogenous spread, malignant melanomas frequently metastasize to the heart; consequently, the tumor has the highest rate of cardiac metastases (in more than half the cases) [15,16].

Myxoma is the most common PCT, with a prevalence of 58.14% among other PCTs, and North America and Europe present the highest rates of PCTs [4,8,17]. The number of female patients with myxoma was observed to be higher than that of male patients [11].

Rhabdomyoma and fibroma are most common among children [12,18]. In pediatric ages, it is also common to find cardiac masses in association with some genetic syndromes (e.g., myxoma in the Carney complex) [19].

## 3. Clinical Presentation

Signs and symptoms are nonspecific and highly variable depending on the location, size, composition, and histopathology of the cardiac mass.

Cardiac masses may cause symptoms through a variety of mechanisms, such as embolization, the obstruction of the circulation through the heart or heart valves, interference with the heart valves (causing regurgitation), the direct invasion of the myocardium (resulting in impaired left ventricular function, arrhythmias, heart block, or pericardial effusion with or without tamponade), the invasion of the adjacent lung that may cause pulmonary symptoms and mimic bronchogenic carcinoma, or constitutional or systemic symptoms [4,6].

## 4. Localization

Generally, most cardiac masses involving the right atrial wall should be suspected of being malignant; the right atrial wall is an uncommon location for benign tumors, although it is a common location for thrombi. Myxomas are typically solitary, vary in size from 1 to 15 cm, and have a predilection for the interatrial septum near the fossa ovalis; approximately 75% of them occur in the left atrium, 20% in the right atrium, and 5% in either ventricle [20].

## 5. Clinical Pills

Performing an accurate anamnestic evaluation is important. Factors such as previous myocardial infarction with severe dysfunction, atrial fibrillation, or the presence of a central venous catheter may suggest thrombotic formation, the most common cardiac mass. Similarly, a history of previous malignancy may suggest the possibility of cardiac metastasis [4].

## 6. Diagnostic Imaging

Although ultrasound remains the first-line imaging technique for visualizing cardiac masses and often allows the discovery of the masses, it often does not allow their characterization [21,22]. The use of transesophageal ultrasound certainly yields information, as in the case of the evaluation of valve rapports, but it remains invasive for the patient [23]. Advanced applications such as 3D and contrast can improve echocardiographic assessment, although multimodal imaging often allows for a better assessment [7,21,24]. Technologically-advanced radiological imaging with CT and CMR is often employed as the second-line approach toward characterizing cardiac masses [3,21,25,26,27,28]. CMR, in particular, allows for characterizing the tissues, with the different MR sequences helping in the diagnosis by looking for specific characteristics of the various tumors [29,30,31,32]. CT can be used to produce detailed images of the heart and surrounding structures. For this reason, CT is usually used as a complementary method to evaluate the relationships with the various cardiac (e.g., coronary) and extracardiac structures and can demonstrate the presence of calcium [3,33]. A PET/CT scan can be used to evaluate the metabolic activity of a cardiac mass, which can offer valuable information about the likelihood of malignancy. Therefore, PET/CT can be particularly useful in the evaluation of suspected cardiac tumors [10]. In addition, the association of the PET study is particularly useful in metastatic locations that include the heart [34]. Chan et al. reported a PET/CT sensitivity and specificity for cardiac neoplasm of 70% and 78%, respectively, but PET/CT sensitivity decreases for “pseudotumors,” such as intracardiac thrombus [35]. Although studies in literature demonstrated that malignant cardiac tumors usually showed a significantly higher metabolism than benign cardiac lesions, further larger studies are needed to confirm these data [36].

## 7. Cardiac Magnetic Resonance

CMR offers a lot of advantages in the evaluation of cardiac masses due to its excellent tissue characterization and multiplanar imaging capabilities. MRI can help determine the size and location of the mass and provide information about the extent of involvement of the surrounding structures; CMR can also evaluate the relationship. In addition, it can evaluate the cardiac function with cine sequences and provide a quantitative objective assessment with T1-T2 mapping. For these reasons, CMR is the preferred imaging modality in the evaluation of cardiac masses [37,38,39] (Table 1).

Standardized protocol needs to be tailored to the specific mass lesion by using optimal imaging planes and saving time-omitting parts of the protocol that may not be relevant for a particular mass.

### 7.1. Bright Blood Imaging

#### 7.1.1. Balanced-Steady-State Free Precession (B-SSFP) Slices of Thorax in Axial, Coronal, and Sagittal Planes

Description: the sequences called true fast imaging (TruFISP), Siemens, fast imaging employing steady-state acquisition (FIESTA), General Electric, and balanced-fast field echo (FFE), Philips, tend to highlight fluids, e.g., blood, by making them appear ‘’bright.’’ The signal intensity is related to the ratio T2/T1, so they are neither T1-weighted (T1w) nor T2-weighted (T2w); in fact, the T2/T1 ratio is small for most solid tissues but not for fat (0.30) and fluids (0.70).

Utility:

- Early localization of the mass and subsequent sequences may be targeted on it
- Panoramic view of the anatomical structures of the thorax

#### 7.1.2. Cine Sequences

Description: cine imaging involves the acquisition of data at multiple time points, known as cardiac phases, throughout the cardiac cycle. They require very short repetition times to be used and therefore can only be achieved using gradient echo-based pulse sequences. The main type of gradient echo pulse sequence used for cine imaging is B-SSFP. B-SSFP is a gradient echo (GRE) sequence that primarily relies on steady-state magnetization for signal production. If the repetition time (TR) is short, residual transverse magnetization will be present during subsequent excitations, eventually leading to the evolution of steady-state magnetization. Cine imaging can also be obtained with spoiled steady-state free precession (sSSFP) with an inferior contrast between myocardial and blood compared with bSSFP. If the patient cannot hold her breath or has multiple intruding arrhythmias, real-time cine imaging can be performed. The procedure comprises a group of single-shot echo-planar methods that allow for the acquisition of bright blood cardiac images in less than 1 *s* per slice without the requirement to hold one’s breath (considerably lower spatial resolution).

- Two-chamber (2CH SSFP): along a plane passing between the center of the mitral-tricuspid annulus and the apex of the left and right ventricles, depending on the location of the tumor.
- Four-chamber (4CH SSFP): along an image plane passing through the center of the left ventricular cavity and the right ventricular costophrenic angle
- Three-chamber (3CH SSFP): along a plane passing between the aortic and mitral annulus and the apex of the left ventricle
- Short-axis (SAX SSFP) acquisitions are acquired along an image plane perpendicular to the interventricular septum, passing through both ventricles and sometimes the atria.

All 2CH, 4CH, 3CH, and SAX stack SSFP sequences should be targeted specifically to encompass the tumor mass.

Utility:

- To evaluate motion and myocardial contraction
- To assess border, size, and location
- Very useful for stable anatomical relationships of the mass with myocardium (intramyocardial or pedunculated) and with adjacent structures
- To evaluate mobile masses, such as atrial myxomas or papillary valve fibroelastoma
- To reveal pericardial effusion
- Cardiac valve function and visualization of stenotic or regurgitant flow jets

**Figure 1:**
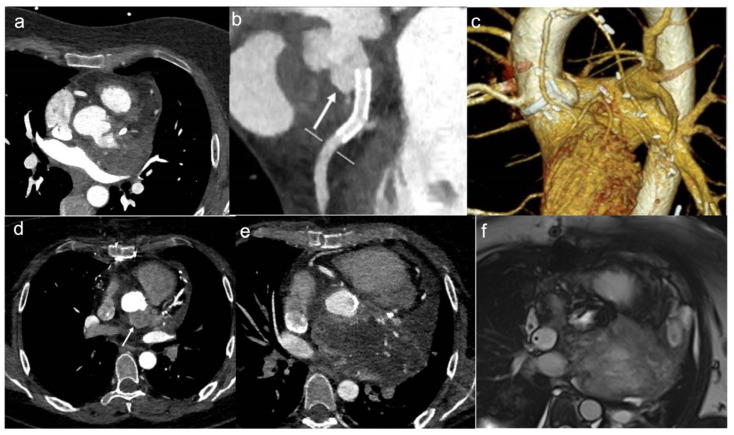
A 46-year-old woman with retrosternal pain during exertion and non -ST elevation myocardial infarction underwent coronarography. The interventional procedure showed stenosis of the common trunk treated with bifurcation stent implantation. Later, due to the unexplained pericardial effusion, the patient underwent coronary computed tomography angiography (CCTA) which revealed a voluminous pseudoaneurysm (arrow) of the left coronary sinus (**a**,**b**). Cardiac surgery was performed to exclude pseudoaneurysm and to perform a by-pass (**c**). After the cardiac surgery, the patient performed a CCTA that showed the exclusion of the pesudoaneurysm (**d**). Four months later, when the patient was back for a follow-up, CT angiography showed a mass with peripheral enhancement, central hypodensity due to necrosis, and central neoangiogenesis (**e**). Finally, the patient underwent CMR that confirmed the presence of inhomogeneous mass invading the surrounding structures (**f**) and neoplasia was suspected. Lastly, the ultrasound-guided transesophageal biopsy has shown the presence of fused and pleomorphic malignant cells with leiomuscular immunophenotype (actin 1A4 +) and the diagnosis of leiomyosarcoma was made.

**Figure 2:**
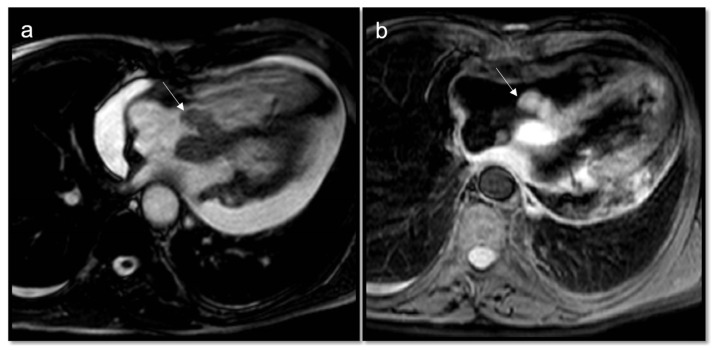
A 78-year-old man arrived at the emergency department with a two-week history of progressively worsening dyspnea, cough, and weight loss. CMR cine sequences show a conspicuous pericardial effusion (**a**) and an ill-defined infiltrative mass (arrow) at the level of the tricuspid valve, hyperintense on T2w Fat Sat (**b**). Biopsy confirmed it as a primary cardiac lymphoma, a very rare entity.

Tips: The border of most benign cardiac tumors is smooth and well-defined, with no irregularity or infiltration [40]. In contrast, malignant cardiac tumors tend to have more lobular, ill-defined, and invasive borders and may invade the myocardium, pericardium, and adjacent extracardiac structures (Figure 1) [41]. Some benign tumors, such as myxomas, have a thin pedicle attaching their border to the myocardium, whereas malignant tumors are usually broad-based [42]. Most benign tumors are small in size compared with malignant tumors, which are usually larger than 5 cm and often in the form of multiple lesions (Figure 2) [43].

### 7.2. Black Blood Imaging

Black blood imaging is predominantly used to evaluate anatomy and characterize the T1 and T2 properties of tissue, and it is referred to as ‘’black blood’’ because double-inversion pulse, fast-flowing blood in the imaged slice appears black. Black blood images can be obtained with spin-echo (SE), turbo spin-echo (TSE), or fast spin-echo (FSE) pulse sequences, with echo and repetition times that have been chosen to create T1w or T2w images. The fast acquisition time of the sequences minimizes respiratory and cardiac movement artifacts. However, a low signal to noise ratio (SNR) results in inferior spatial resolution.

#### 7.2.1. T2-Weighted Triple-Inversion Recovery Images

Description: water has a long T2 and will therefore show up more brightly. Usually, T2-weighted sequences including fat suppression are acquired. There are two main methods to this end [44].

1. Short tau inversion recovery (STIR) [40]: STIR is a robust method of fat suppression. Inversion recovery (IR) is the most used method of magnetization preparation. IR depends on the fact that different tissues have different T1 characteristics. If the time between inversion and imaging (TI) is chosen carefully, the signal from a given tissue can be eliminated. STIR relies on the short T1 of fat compared with other tissues. Therefore, the fat magnetization will pass through the null point of an IR sequence before the tissue of interest. If imaging is carried out at the null point of fat, the signal from the fat will be suppressed.
2. Spectral inversion recovery (SPIR): spectral-selective pulses rely on the fact that water and fat precess at slightly different frequencies (approximately 220 Hz difference at 1.5T). Therefore, a special RF pulse that only excites fat can be utilized. In SPIR, a spectrally selective 180° pulse is used to invert only the fat magnetization. The water magnetization is unchanged by the spectrally selective 180° pulse. The fat magnetization is then allowed to recover, and a TI that coincides with the null point of fat is chosen. Unlike STIR, at the onset of imaging, all the water magnetization is in the longitudinal axis, and therefore, there is no loss in SNR.

**Figure 3:**
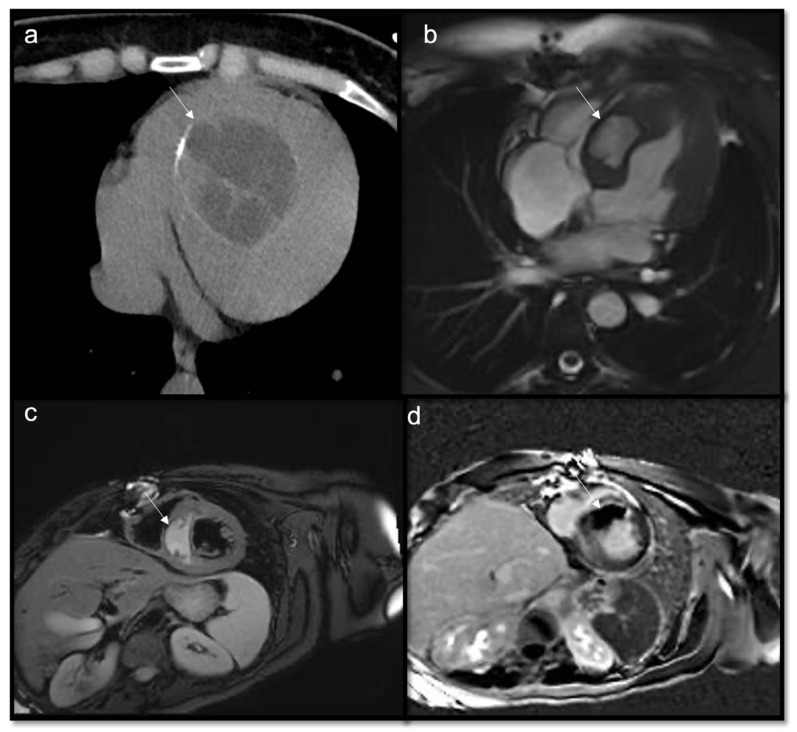
Cardiac hydatidosis (arrow) revealed in CT (**a**); CMR cine sequences showing a cyst within the septum (**b**), as confirmed in T2w Fat Sat (**c**), without late gadolinium enhancement (**d**). The standard treatment for cardiac hydatidosis is surgery as early as possible even if there is a high risk of the cyst rupturing inside the heart chamber.

Utility:

- High signal: fluid, myxomatous components
- Low signal: calcific components
- To recognize fluid content such as a pericardial cyst or myocardial cyst (Figure 3)
- To assess for edema or necrosis in the mass.

#### 7.2.2. T1-Weighted Double-Inversion Recovery Images

Utility:

- To achieve a better anatomic definition than T2w ones
- High signal: lipid content, melanin, blood catabolites (methemoglobin)
- Low signal: indicates fibrosis

Red Flag (!): Except for myxoma, which may contain cysts, regions of necrosis, fibrosis, hemorrhage, and calcification, heterogenous T1w and T2w signal intensity patterns, reflecting tumor tissue necrosis or hemorrhage which suggests malignancy, as well as the presence of hemorrhagic pericardial effusion.

Tip: Differentiating lipoma from lipomatosis of interatrial septum

**Figure 4:**
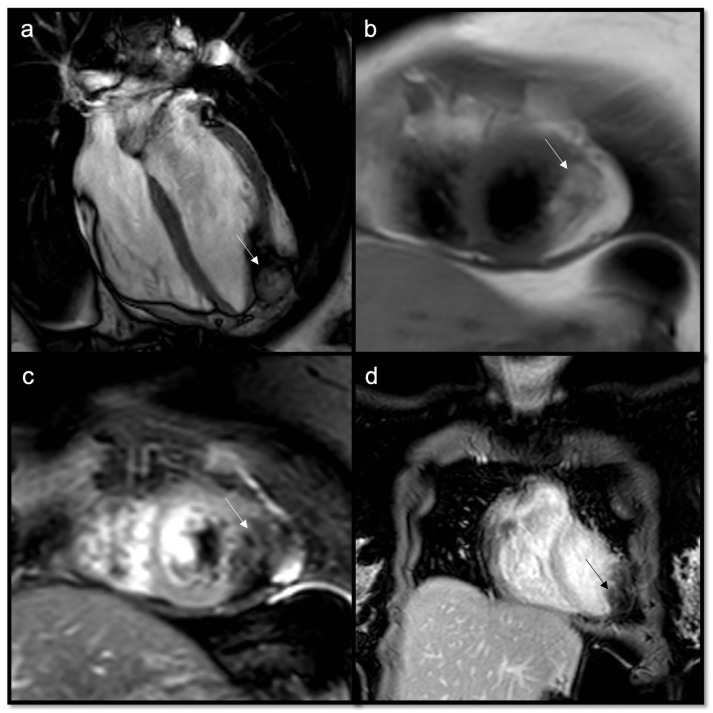
Apical intramyocardial lipoma (arrow) appears as a well-defined mass, capsulated with a chemical shift dark rim in cine images (**a**), hyperintensity on T1w due to fat content (**b**), signal loss on T2w Fat Sat sequences (**c**), and no enhancement in post-contrast sequences (**d**).

**Figure 5:**
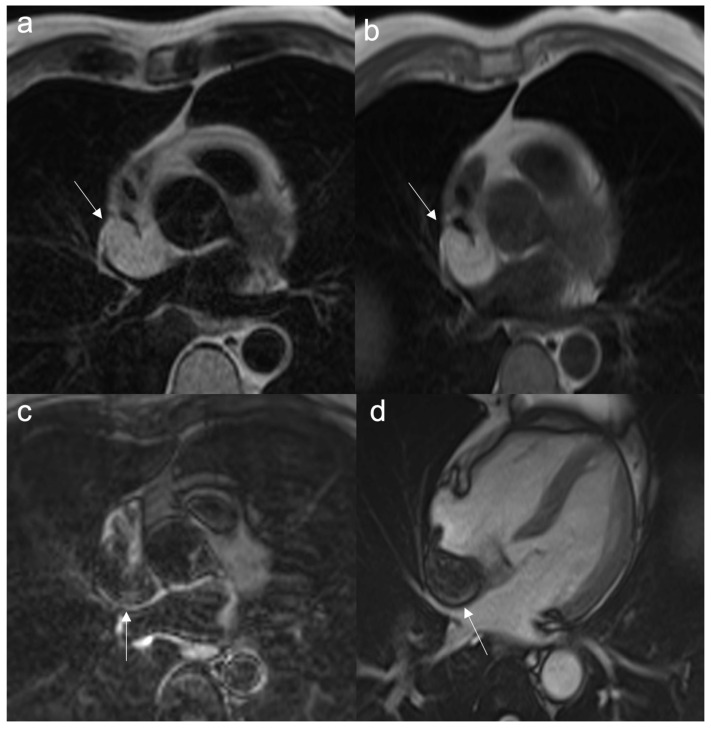
Lipomatous hypertrophy of the interatrial septum (arrow): a 70-year-old woman with acute chest pain arrived at the Emergency Department; a CT scan shows a lung mass suspected of cancer and a hypodense lobulated mass, with densitometric values similar to lipid, extending from the atrial septum upward to the outlet of the SVC in the right atrium. The mass is hyperintense on both MRC T1w and T2w sequences (**a**,**b**), has signal loss in T2w Fat Sat due to its lipid content (**c**), and appears in a typical “dumbbell’’ shape in 4CH SSFP sequences (**d**).

True cardiac lipomas are encapsulated, contain neoplastic fat cells, and occur in young patients (Figure 4). Lipomatous hypertrophy of the interatrial septum is often found in older and overweight patients. Unlike true lipomas, they are not capsulated and contain lipoblasts and mature fat cells. Because of the presence of brown fat, this entity may show an increased radiotracer uptake on PET/CT scans. The bilobed, dumbbell-shaped fatty mass must be larger than 20 mm in thickness and characteristically spare the fossa ovalis (Figure 5). It is important to recognize this in patients undergoing percutaneous endovascular procedures involving transseptal puncture. The key diagnostic finding on CMR images indicates homogeneous high-signal intensity (relative to the myocardium) on T1-weighted images that markedly suppresses with fat saturation. Both conditions are avascular and do not enhance with contrast material.

#### 7.2.3. First-Pass Perfusion Sequences

Description: they are T1w saturation recovery gradient echo pulse sequences where gadolinium contrast is administered and imaged over >40 heartbeats.

Utility:

- Assessment of vascularity
- Malignant masses often show heterogeneous enhancement; typically, angiosarcoma shows early avid enhancement as it is richly vascularized.
- Trombi due to their avascular nature appear non-enhanced.

#### 7.2.4. Early Gadolinium Enhancement (EGE)

Description: it is an IR gradient echo sequence that is performed 1–3 min after contrast administration.

Utility:

It is the best sequence to evaluate suspect of thrombus; in this case, a long TI of ~440 ms is chosen to null any non-vascular lesions (e.g., thrombus) that do not contain gadolinium.

Tip: Distinguishing a myxoma from a thrombotic formation

**Figure 6:**
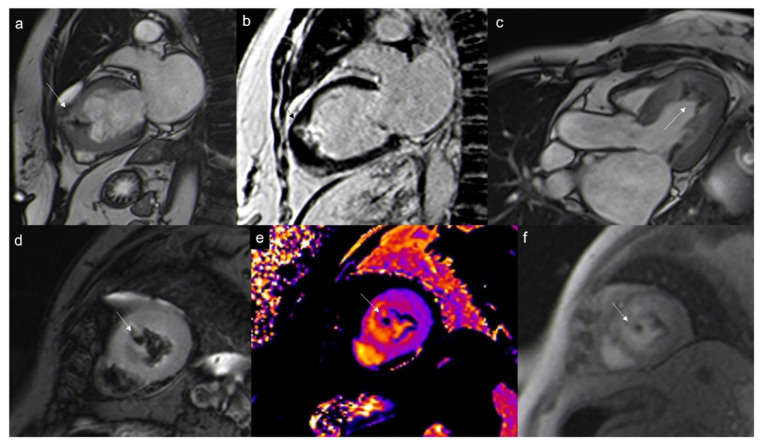
Chagas Disease in a 67-year-old woman. The 2CH and 4CH (**a**,**c**) views demonstrate an LV apical aneurysm with intra-cavitary thrombi (arrows). Thrombi do not show enhancement on LGE images (**b**) and appear hypointense on T2 weighted sequence (**d**) with very low T1 native value (**e**). It shows no enhancement on first pass perfusion sequences due to their avascular nature (**f**).

**Figure 7:**
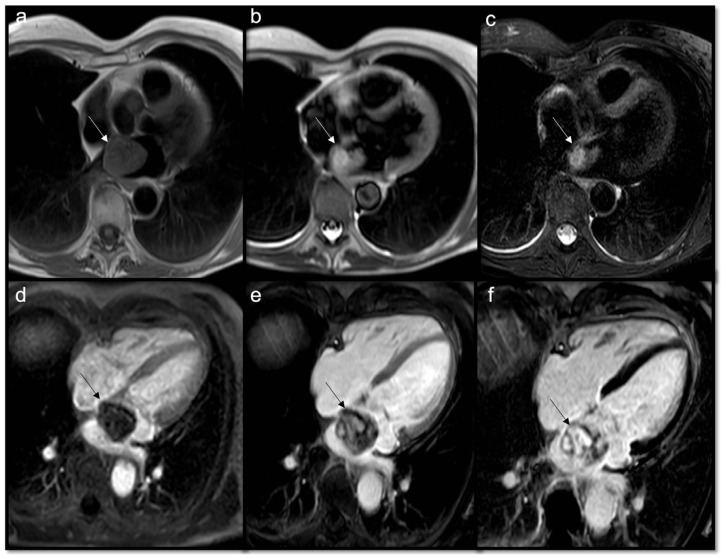
Typical myxoma (arrow) appears as a well-defined, smooth, oval, or lobular lesion, has an intermediate signal in T1w (**a**), heterogeneous hyperintensity in T2w and T2w Fat Sat (**b**,**c**), linked to the high presence of water in the myxoid stroma. It shows limited enhancement on first-pass perfusion sequences (**d**) and progressive heterogeneous enhancement on late gadolinium enhancement (LGE) sequences due to its fibrous content (**e**,**f**). Some cases may show hyperintensity on T1w images due to hemorrhagic foci. Cine imaging is of particular value in this case as these lesions can be highly mobile and prolapse through the atrioventricular valves during diastole, causing temporary obstruction to blood flow.

Classically in front of an atrial mass, the two main differential diagnoses are myxoma and thrombus. T1w sequences alone are not enough, and T2w can be misleading. In fact, thrombi in the very acute phase can show T2 hyperintensity (due to oxyhemoglobin), and the progressive decrease of T2 times begins only after a few weeks. Post-contrast sequences and particularly EGE increase the accuracy in the distinction, as a thrombus does not show internal vascularization (Figure 6). Myxoma, instead, shows progressive and inhomogeneous enhancement linked to the presence of fibrous stroma (Figure 7) [45].

Drawbacks:

- Organized thrombi may show peripheral enhancement on LGE images, owing to their fibrous content.
- T1w and T2w signal characteristics vary depending on the age of a thrombus. Thrombi generally showed T1 values similar to those of the normal myocardium, with a significant difference between recent (shorter T1) and old (longer T1) thrombi. The T2 relaxation times of thrombi were consistently longer than myocardial T2, regardless of their age [46]. Most of the literature reviews classify thrombi according to their age as acute, subacute, or chronic. Theoretically, at the very acute phase of their formation, thrombi are usually T1- and T2-hyperintense (short T1 and long T2) because hemoglobin is still oxygenated. In a subacute thrombus, hemoglobin is metabolized into methemoglobin. The paramagnetic effect of methemoglobin (shortening of the relaxation times) is responsible for a high T1 signal (shortening of T1). The T2 signal is generally increased (longer T2) because of water content due to red cell lysis. After a longer period, the thrombus is depleted of water, and cell debris containing methemoglobin is replaced by fibrous tissue, responsible for a decrease of signal on T1-weighted images (longer T1) and a decrease of signal on T2-weighted images (shortening of T2) [47,48].

#### 7.2.5. Late Gadolinium Enhancement (LGE)

Description: it is a T1w sequence acquired 10 min after the contrast injection. Because gadolinium shortens T1, it results in an enhancement of the area with a major contrast medium accumulation [49].

The imaging usually has a high spatial resolution and covers the whole heart, using multiple contiguous slices. A single slice is acquired per breath hold, and the whole heart is obtained after several breath holds; hence, the breath-hold times limit the spatial resolution that can be achieved [50].

LGE is a sequence based on the kinetics of the gadolinium itself. Gadolinium is a paramagnetic contrast agent that has a typical extracellular distribution and timing of persistence. When injected intravenously, if myocardial tissue is normal, it remains for 10–20 min among the cells before it is washed away. Whenever there is a pathological process, the kinetics vary, and the wash-out is reduced, resulting in longer persistence of the myocardial tissue [51]. This is due to every process that causes a decreasing ratio of normal myocytes to extracellular space, such as the ruptures of the membranes’ cells, as in an infarcted lesion, or a process that expands the interstitial space such as tissue fibrosis or inflammation [52].

**Figure 8:**
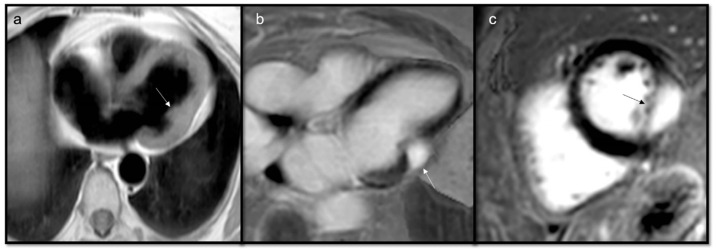
Fibroma (arrow): a suspected infiltrative intramural mass at the lateral wall of left ventricle is seen in a 70-year-old woman with hypertension, isointense to myocardium on T1w (**a**). Because of the hypovascular and fibrotic nature of this lesion, it is typically hyperintense and homogeneous on LGE, as shown in 4CH and SA slices (**b**,**c**). Sometimes fibromas may have central calcifications with the blooming of calcific components in GE sequences.

**Figure 9:**
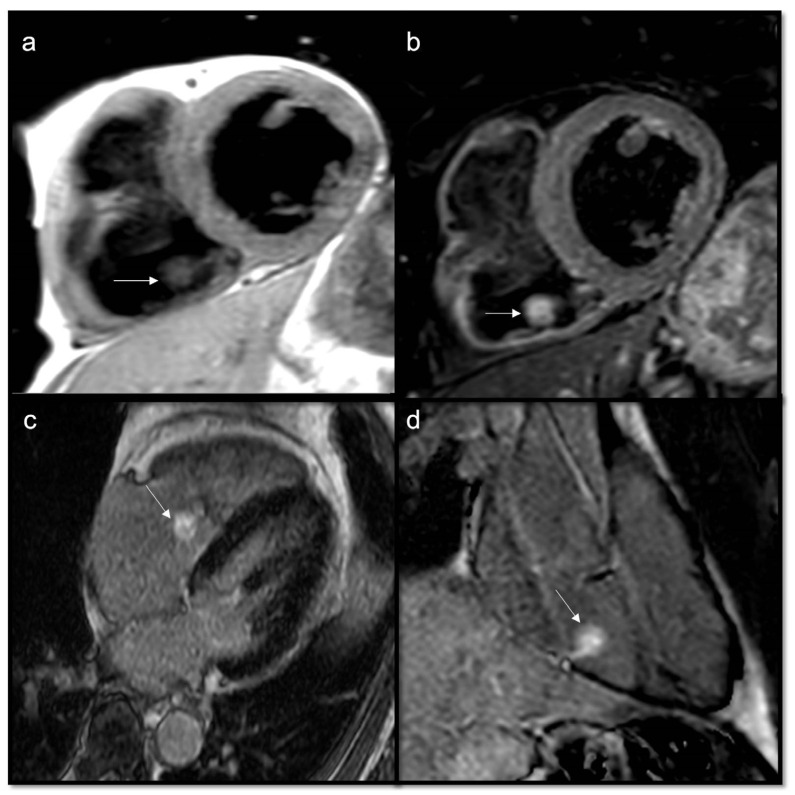
Papillary fibroelastoma of the tricuspid valve (arrow) appears as a highly mobile, spherical pedunculate mass attached to the posterior tricuspid valve leaflet and on T1w images (**a**) it is homogeneous; no fatty content is found in T2w Fat Sat (**b**). LGE images demonstrate a hyperintense signal caused by fibroelastic tissue of the mass in 4CH and 2CH of the right ventricle (**c**,**d**). Approximately 80% occur concerning either the aortic or mitral valve, while tricuspid and pulmonary involvements are much rarer.

**Figure 10:**
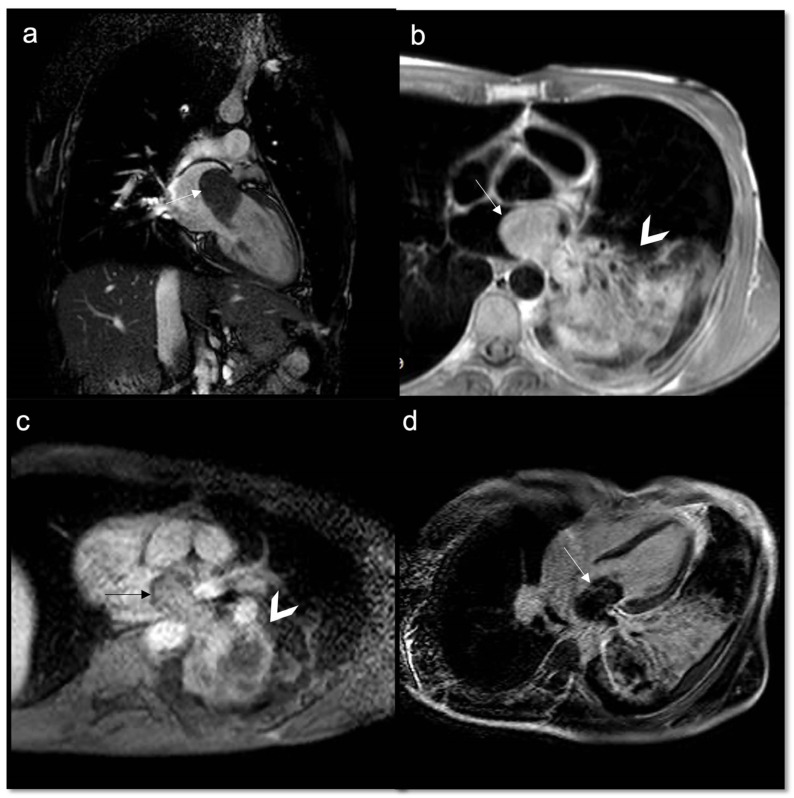
Neuroendocrine lung tumor with left atrial metastasis due to contiguous spread (**a**); the atrial mass (arrow) has the same signal as the lung malignant tissue (arrow head) in axial T1w sequences (**b**), shows heterogeneous enhancement in EGE SA (**c**), and indicates a slight enhancement in LGE 4CH (**d**).

**Figure 11:**
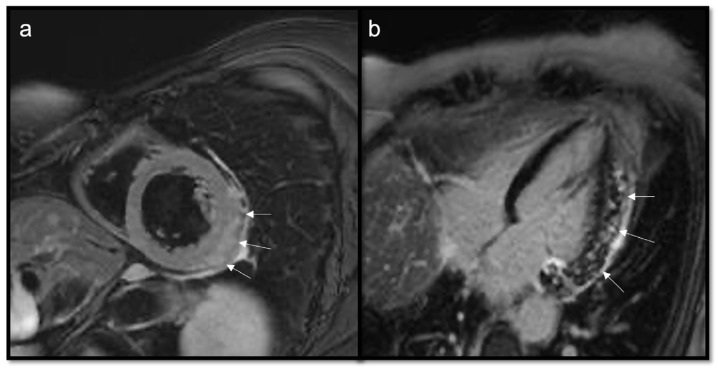
Multiple epi- and intra-myocardial nodules (arrows) along the lateral wall of the left ventricle; subtle hyperintensity on T2w Fat Sat (**a**) that enhances in LGE (**b**) in a patient with thymoma.

**Figure 12:**
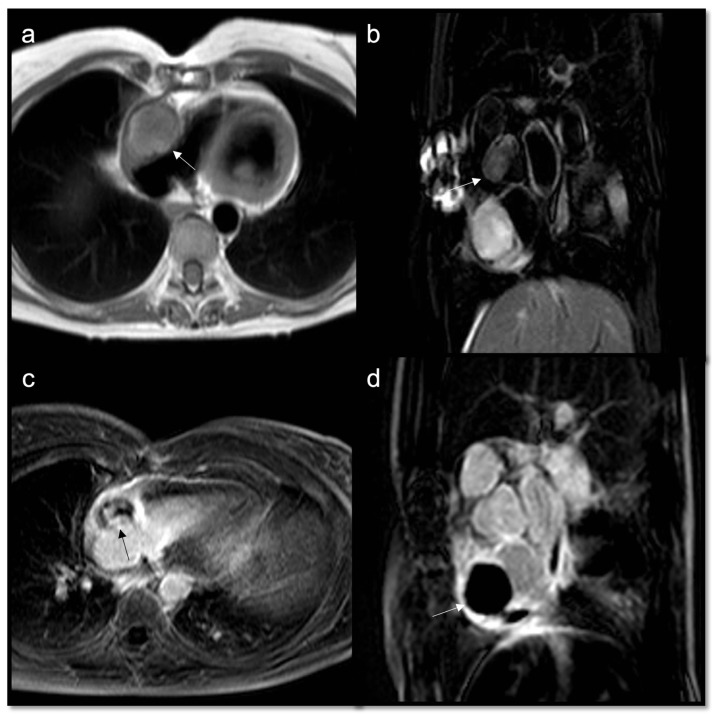
Heterogeneous right atrial mass (arrow) iso-hypointense in T1w and slightly hyperintense in T2w Fat Sat (**a**,**b**), with inhomogeneous enhancement in LGE axial acquisition (**c**), turned out to be an angiosarcoma. After multiple cycles of chemotherapy and radiotherapy, there was no perceptible enhancement in LGE SAX acquisition (**d**).

**Figure 13:**
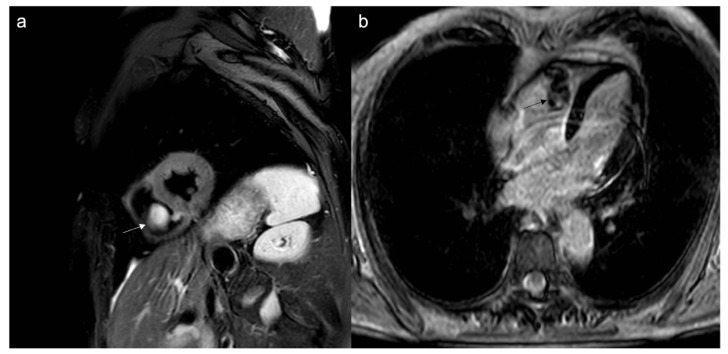
Rhabdomyosarcoma (arrow) is hyperintense on T2-weighted images (**a**) and it shows contrast enhancement with regions of hypointensity due to central necrosis (**b**).

Using an appropriate TI, after the first 180° impulse, allows the nulling of the signal of normal myocardiocites, which appear black, whereas the pathological myocardium with LGE results appear white (Figure 8, Figure 9, Figure 10, Figure 11, Figure 12 and Figure 13). To obtain an appropriate TI, we can use a preventive look-locker sequence that acquires images at different TI. Anyway, we can use a phase-sensitive inversion recovery (PSIR) sequence that is less sensitive to TI variations [53].

#### 7.2.6. T1 and T2 Mapping

Mapping is an MRI technique used to calculate the T1 or T2 time of a certain tissue and display them voxel by voxel on a parametric map, helping in myocardial tissue characterization [54].

Description: native T1 refers to the T1 time measured in the absence of a contrast agent, is related to the water, protein, lipid, and iron content of the respective tissue, and it expresses the signal from the intracellular and extracellular compartments.

Postcontrast T1 measured after the application of gadolinium is used to calculate extracellular volume (ECV), a surrogate parameter for the extracellular matrix.

The main types of sequences for T1 mapping include the following:

- Using inversion preparation
- Modified look-locker imaging (MOLLI) is the most widely used T1 measurement sequence. In 5 (3), MOLLI measurements are obtained at different TIs over 5 + 3 = 8 heartbeats with a 3-beat recovery period in between, while in the post-contrast sequences, a 4 (3) 3 scheme is used due to shortener T1 times.
- Shortened MOLLI (SHMOLLI) is used for patients who cannot hold their breaths for more than 20 s; shortened MOLLI (shMOLLI) using a 5–1–1 scheme is available.
- Using saturation preparation
- Saturation-recovery single-shot acquisition (SASHA) recovery methods use 90° instead of 180° pulses, which have the advantage of removing magnetization memory from prior cycles and allowing a direct estimate of THE true T1 to be made. Disadvantages include lower SNR and more artifacts [55].
- Using a combination of inversion and saturation
- Saturation-pulse prepared heart-rate independent inversion-recovery (SAPPHIRE) uses a hybrid of saturation and inversion pulses to improve the precision of the saturation-recovery approach while maintaining accuracy.

In patients with atrial fibrillation, systolic data acquisition might be more robust than diastolic readout; in that case, we can find lower T1 values [56].

The main types of sequences for T2 mapping include the following:

- T2 turbo spin multi-echo (T2-TSE)
- T2 prepared steady-state free precession (T2p-SSFP)
- T2 gradient spin-echo mapping sequences (T2-GraSE) [57]

T2 mapping can be conducted with one of the above sequences, and at the same time, a series of co-registered images can be acquired with different T2 echo times. Then T2 values can be computed pixel-wise from a signal intensity versus echo time curve fitting model. The respective voxels can then be quantified and evaluated, based on normal reference values in diffuse disease or by comparing them to the spared healthy myocardium in focal disease. Prolonged T2 reflects myocardial edema, allowing a more objective quantification of myocardial edema than standard black-blood T2W STIR images, which are often limited by susceptibility or motion artifacts.

Both normal values of native T1 and T2 maps, but mainly the T1 ones, change based on magnetic field strength (1.5 or 3 tesla) and the type of sequence; therefore, a local reference range is highly recommended [58]. T2 values tend to decrease at a higher magnetic field strength, as opposed to T1 values, which tend to increase [59].

Utility:

Parametric mapping is very useful in patients undergoing evaluation for suspected myocardial disease, and it is also beneficial for the evaluation of cardiac masses. Recent studies evaluating T1 and T2 mapping suggest that parametric mapping increases the ability of CMR to differentiate between cardiac thrombi and masses [58,60,61,62,63]. The analysis of intracardiac tumors and masses with T1 and T2 mapping revealed important differences among the different subtypes, generating multiple T1/T2 profiles according to the etiology [46]:

- Short T1/short T2 (as compared with the myocardium) for calcifications
- Short T1/long T2 for melanoma or lipomas and lipomatosis [64]
- Long T1/long T2 for most tumors, whether benign or malignant, with different degrees in particular for the T1 (close to the myocardium for rhabdomyoma, long or very long for myxomas [65] and fibroelastomas)
- T1 mapping allows for highlighting the difference between recent (<1 week) and old thrombi (>1 month), as recent thrombi have a significantly shorter T1.

Caspar et al. did not observe any differences concerning T2 mapping sequences in their cohort, and T2 is long compared with the myocardium, regardless of the age or location of the thrombus. This might be explained because it is very uncommon to diagnose a thrombus in a very acute phase when T2 is still long.

Red Flag (!): Heterogenous maps due to hemorrhage or necrosis inside the mass suggest malignancy.

#### 7.2.7. Extracellular Volume

Description: post-contrast T1 mapping allows for the calculation of ECV using hematocrit, native T1 relaxation times of myocardium, and blood pool. If the real value of hematocrit is not available (the use of a value found a few hours before the exam is suggested), synthetic hematocrit derived from the longitudinal relaxation of blood (formula varies with machine and sequence type) can be used [66].

**Figure 14:**
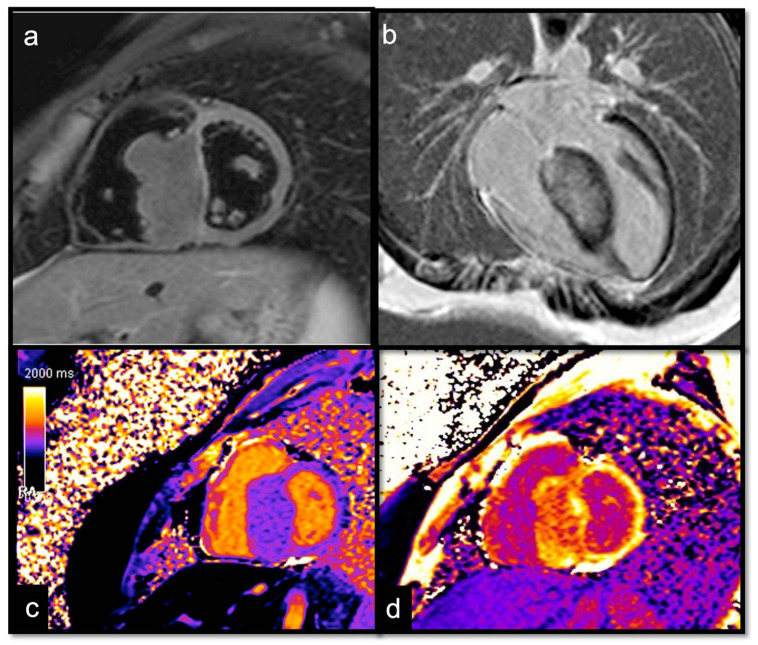
Suspected HCM in a 14-year-old child, with asymmetric hypertrophy of the left ventricle. In T2w, the septal signal is very different from other myocardial segments (**a**), and it shows intramyocardial LGE (**b**), with a slight increase in T1 native values, approximately 1000 ms (normal value < 950 ms in our site) (**c**) and increased ECV around 41% (normal value < 30%) (**d**). The diagnosis was overturned and confirmed as a fibroma.

**Figure 15:**
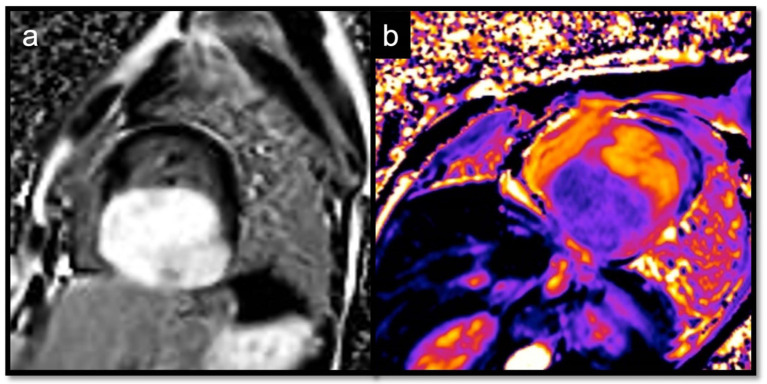
Another case of intramyocardial fibroma in a 5-year-old child, involving inferior-lateral and inferior-septal segments of the left ventricle, mimicking HCM; the mass shows an intense and homogenous LGE (**a**), with normal-low T1 native values (**b**), but a very high ECV, around 80%.

Utility:

- ECV is elevated in conditions associated with the expansion of the myocardial interstitium, such as fibrosis or infiltration [67,68].
- ECV can be of help in differentiating hypertrophic cardiomyopathy (HCM) from other conditions that may mimic it (Figure 14 and Figure 15) [69].

#### 7.2.8. Phase Contrast (PhC) Sequences

Description: use bipolar gradients and typically are acquired in a plane perpendicular to blood flow.

Utility: in case of valve involvement, PhC sequences might be performed for the quantitative assessment of the hemodynamic effect of the mass.

## 8. Conclusions

CMR is the gold standard for the noninvasive characterization of cardiac masses, allowing for better tissue characterization compared with echocardiography and a better assessment of the relationship between tumors and adjacent structures to guide surgical resection. Finally, it provides an accurate assessment of cardiac function and any hemodynamic involvement.

## Figures and Tables

**Table 1:** Most common cardiac tumors.

Tumor in Order of Frequency	Steady-State Free Precession	T2W	Fat Sat	T1W	First-Pass	LGE
*Benign*						
*Mixoma*	mobile, pedunculated	hyper	-	iso	+	+ heterogeneous
*Fibroma*		ipo	-	iso	-	+ homogeneous
*Lipoma*	chemical shift dark rim	hyper	signal loss	hyper	-	none
*Hemangioma*	hyper	hyper	-	iso	+	+ homogeneous
*Malign*						
*Metastases*	heterogeneous	iso-hyper	-	iso, ipo, or hyper (melanin, blood)	+	+
*Angiosarcoma*	heterogeneous iso	heterogeneous hyper	-	heterogeneous hyper	++ avid “sunray” aspect	++ heterogeneous
*Lymphoma*	heterogeneous iso	slightly hyper	-	iso	+	+ heterogeneous

Hyper, ipo, and iso stand for hypertintense, ipointense, and isointense, respectively. ++ is for “very”.

## Data Availability

No new data were created or analyzed in this study. Data sharing is not applicable to this article.
